# Supplementary material for: Modeling glioblastoma heterogeneity as a dynamic network of cell states
Source: Mol Syst Biol. 2021 Sep 16;17(9):e10105. doi: 10.15252/msb.202010105 (PMC8444284; doi:10.15252/msb.202010105)
Supplement: Supplementary file 6 — Source Data for Figure 5 [file MSB-17-e10105-s004.zip › Figure5A_sourcedata/GSEA_3071/hallmarks_stateV.GseaPreranked.1623742546329/HALLMARK_MYOGENESIS.html]

Details for gene set HALLMARK\_MYOGENESIS[GSEA]

|  || Dataset | state43071 |
| Phenotype | NoPhenotypeAvailable |
| Upregulated in class | na\_neg |
| GeneSet | HALLMARK\_MYOGENESIS |
| Enrichment Score (ES) | -0.4500671 |
| Normalized Enrichment Score (NES) | -2.289371 |
| Nominal p-value | 0.0 |
| FDR q-value | 0.0 |
| FWER p-Value | 0.0 |
Table: GSEA Results Summary

  

Fig 1: Enrichment plot: HALLMARK\_MYOGENESIS      
 Profile of the Running ES Score & Positions of GeneSet Members on the Rank Ordered List

  

| PROBE | GENE SYMBOL | GENE\_TITLE | RANK IN GENE LIST | RANK METRIC SCORE | RUNNING ES | CORE ENRICHMENT || 1 | CKB |  |  | 23 | 1.748 | 0.0210 | No |
| 2 | ITGA7 |  |  | 37 | 1.644 | 0.0425 | No |
| 3 | IGFBP3 |  |  | 78 | 1.437 | 0.0557 | No |
| 4 | NCAM1 |  |  | 117 | 1.334 | 0.0679 | No |
| 5 | PKIA |  |  | 138 | 1.297 | 0.0829 | No |
| 6 | CRYAB |  |  | 176 | 1.221 | 0.0936 | No |
| 7 | CDH13 |  |  | 185 | 1.204 | 0.1096 | No |
| 8 | CHRNA1 |  |  | 346 | 1.021 | 0.0939 | No |
| 9 | CDKN1A |  |  | 363 | 0.993 | 0.1053 | No |
| 10 | NAV2 |  |  | 409 | 0.944 | 0.1104 | No |
| 11 | CLU |  |  | 464 | 0.900 | 0.1131 | No |
| 12 | EPHB3 |  |  | 498 | 0.868 | 0.1195 | No |
| 13 | APP |  |  | 570 | 0.818 | 0.1178 | No |
| 14 | NOTCH1 |  |  | 673 | 0.762 | 0.1093 | No |
| 15 | SORBS1 |  |  | 763 | 0.717 | 0.1027 | No |
| 16 | RB1 |  |  | 810 | 0.696 | 0.1041 | No |
| 17 | KCNH2 |  |  | 955 | 0.638 | 0.0858 | No |
| 18 | MRAS |  |  | 1108 | 0.590 | 0.0653 | No |
| 19 | EFS |  |  | 1120 | 0.588 | 0.0717 | No |
| 20 | CHRNB1 |  |  | 1125 | 0.586 | 0.0795 | No |
| 21 | MYL6B |  |  | 1411 | 0.501 | 0.0323 | No |
| 22 | ERBB3 |  |  | 1521 | 0.477 | 0.0183 | No |
| 23 | WWTR1 |  |  | 1794 | 0.419 | -0.0276 | No |
| 24 | REEP1 |  |  | 1905 | 0.403 | -0.0428 | No |
| 25 | MAPRE3 |  |  | 1995 | 0.390 | -0.0542 | No |
| 26 | LARGE1 |  |  | 2006 | 0.387 | -0.0504 | No |
| 27 | SCHIP1 |  |  | 2028 | 0.383 | -0.0489 | No |
| 28 | DAPK2 |  |  | 2285 | 0.351 | -0.0928 | No |
| 29 | SORBS3 |  |  | 2361 | 0.341 | -0.1022 | No |
| 30 | CRAT |  |  | 2458 | 0.327 | -0.1158 | No |
| 31 | HSPB2 |  |  | 2875 | 0.282 | -0.1913 | No |
| 32 | SH2B1 |  |  | 2884 | 0.282 | -0.1887 | No |
| 33 | SYNGR2 |  |  | 2933 | 0.276 | -0.1939 | No |
| 34 | PLXNB2 |  |  | 3068 | 0.262 | -0.2157 | No |
| 35 | AKT2 |  |  | 3094 | 0.260 | -0.2167 | No |
| 36 | LAMA2 |  |  | 3449 | -0.289 | -0.2802 | No |
| 37 | TPM3 |  |  | 3482 | -0.297 | -0.2821 | No |
| 38 | ITGB1 |  |  | 3614 | -0.317 | -0.3025 | No |
| 39 | BHLHE40 |  |  | 3620 | -0.318 | -0.2988 | No |
| 40 | GSN |  |  | 3695 | -0.331 | -0.3082 | No |
| 41 | SOD3 |  |  | 3708 | -0.332 | -0.3057 | No |
| 42 | KIFC3 |  |  | 3738 | -0.337 | -0.3063 | No |
| 43 | CNN3 |  |  | 3751 | -0.339 | -0.3037 | No |
| 44 | SVIL |  |  | 3763 | -0.342 | -0.3008 | No |
| 45 | KLF5 |  |  | 4064 | -0.401 | -0.3523 | No |
| 46 | FLII |  |  | 4095 | -0.408 | -0.3521 | No |
| 47 | MEF2C |  |  | 4351 | -0.487 | -0.3939 | No |
| 48 | STC2 |  |  | 4358 | -0.488 | -0.3879 | No |
| 49 | MYO1C |  |  | 4421 | -0.508 | -0.3924 | No |
| 50 | IGFBP7 |  |  | 4622 | -0.604 | -0.4219 | No |
| 51 | TEAD4 |  |  | 4732 | -0.665 | -0.4330 | No |
| 52 | HBEGF |  |  | 4822 | -0.734 | -0.4394 | Yes |
| 53 | PDLIM7 |  |  | 4867 | -0.773 | -0.4365 | Yes |
| 54 | PDE4DIP |  |  | 4923 | -0.833 | -0.4349 | Yes |
| 55 | TGFB1 |  |  | 4944 | -0.861 | -0.4262 | Yes |
| 56 | SH3BGR |  |  | 4952 | -0.870 | -0.4149 | Yes |
| 57 | SPHK1 |  |  | 4997 | -0.928 | -0.4098 | Yes |
| 58 | MYH9 |  |  | 5090 | -1.125 | -0.4111 | Yes |
| 59 | LSP1 |  |  | 5113 | -1.190 | -0.3980 | Yes |
| 60 | GADD45B |  |  | 5155 | -1.335 | -0.3864 | Yes |
| 61 | COL15A1 |  |  | 5190 | -1.490 | -0.3712 | Yes |
| 62 | COL4A2 |  |  | 5198 | -1.545 | -0.3501 | Yes |
| 63 | NQO1 |  |  | 5210 | -1.596 | -0.3289 | Yes |
| 64 | TPM2 |  |  | 5247 | -1.967 | -0.3072 | Yes |
| 65 | COL6A2 |  |  | 5265 | -2.276 | -0.2773 | Yes |
| 66 | BIN1 |  |  | 5273 | -2.451 | -0.2430 | Yes |
| 67 | TAGLN |  |  | 5281 | -2.669 | -0.2055 | Yes |
| 68 | SSPN |  |  | 5284 | -2.752 | -0.1658 | Yes |
| 69 | COL3A1 |  |  | 5292 | -3.630 | -0.1143 | Yes |
| 70 | COL6A3 |  |  | 5293 | -3.722 | -0.0601 | Yes |
| 71 | COL1A1 |  |  | 5294 | -4.144 | 0.0002 | Yes |
Table: GSEA details [plain text format]

  

Fig 2: HALLMARK\_MYOGENESIS: Random ES distribution      
 Gene set null distribution of ES for **HALLMARK\_MYOGENESIS**
